# Fatal Aortic Regurgitation in Behçet's Disease: A Case Report Highlighting Pitfalls and Lessons in Preoperative Diagnosis

**DOI:** 10.2174/0115734056421966251114111950

**Published:** 2025-11-29

**Authors:** Juan Wu, Xiaofeng Wang, Fang Nie

**Affiliations:** 1 Department of Ultrasound, Lanzhou University Second Hospital, Lanzhou University, Lanzhou 730030, China

**Keywords:** Behçet’s disease, Aortic regurgitation, Prosthetic valve dehiscence, Perivalvular leakage, Transthoracic echocardiography, Bentall procedure, Bentall procedure

## Abstract

**Introduction:**

Behçet's disease (BD), a chronic multisystem inflammatory disorder, rarely involves the heart. Aortic regurgitation (AR) is the predominant valvular lesion. When AR precedes characteristic mucocutaneous symptoms, misdiagnosis and treatment delays often occur.

**Case Presentation:**

We, herein, report the case of a 37-year-old male presenting with isolated aortic regurgitation (AR) as the initial manifestation of Behçet's disease (BD). Initial echocardiography revealed severe eccentric AR with left coronary cusp prolapse and a vegetation-like lesion, raising suspicion of infective endocarditis; however, relapsed oral ulcers developed postoperatively, ultimately confirming BD diagnosis. Despite successful aortic valve replacement, delayed diagnosis due to absent early mucocutaneous symptoms contributed to catastrophic prosthetic valve dehiscence with severe paravalvular leak. The patient underwent an emergency Bentall procedure with venoarterial extracorporeal membrane oxygenation (VA-ECMO) support but succumbed to cardiogenic shock and multiorgan failure. Pathological analysis demonstrated tissue necrosis with minimal inflammation.

**Conclusion:**

Isolated AR may be BD's initial manifestation, preceding classic symptoms by months. Echocardiographic features, including valve prolapse and perivalvular lesions, despite their non-specificity, should prompt screening for BD. Inherent tissue fragility in BD significantly elevates postoperative risks of paravalvular leak and prosthetic valve dehiscence. Early identification, optimal surgical procedure, and timely immunosuppressive therapy are essential to improve the prognosis of cardiac BD.

## 1 INTRODUCTION

Behçet's disease (BD) is a chronic multisystem inflammatory disorder characterized pathologically by vasculitis, first described in 1937 by the Turkish dermatologist Hulusi Behçet. The disease is defined by the classic triad of recurrent oral ulcers, genital ulcers, and uveitis, with potential involvement of the cardiovascular, neurological, and gastrointestinal systems [1]. Although cardiac involvement (cardiac-Behçet) occurs in only 5%-10% of cases, it manifests as heterogeneous and prognostically grave conditions, including pericarditis, valvular lesions, coronary arteritis, and conduction abnormalities. Critically, 25% of cardiac-Behçet cases initially present with isolated valvular damage, predominantly aortic regurgitation (AR), whereas characteristic mucocutaneous manifestations may appear years later, frequently resulting in diagnostic delay [2]. This report describes a fatal cardiac-Behçet case initially masquerading as infective endocarditis, presenting with isolated AR and complicated by a critically delayed diagnosis. We provide an in-depth analysis of the echocardiographic characteristics, the pivotal dilemma surrounding surgical timing, and subsequent clinical management strategies, highlighting the lessons learned from this tragic outcome.

## 2 CASE REPORT

A 37-year-old male was admitted for hepatobiliary surgery with acute cholecystitis and multiple gallstones. Physical examination revealed a body temperature of 39.0°C, blood pressure of 122/61 mmHg, and a grade IV/VI diastolic blowing murmur at the second left intercostal space. The patient reported occasional chest oppression after activity, corresponding to New York Heart Association (NYHA) class II. Laboratory findings included a white blood cell count of 10.95×10^9^/L (neutrophils 81%), total bilirubin 78 μmol/L, procalcitonin 0.115 ng/mL, C-reactive protein (CRP) 28.5 mg/L, pro-BNP 4692 pg/mL, and troponin I 30 ng/L (reference <14 ng/L). Autoimmune antibodies (ANA, ANCA, anti-dsDNA) were negative. Computed tomography (CT) revealed bilateral pleural effusions with pulmonary infiltrates, while CT angiography ruled out aortic dissection or aneurysm. Transthoracic echocardiography (TTE) revealed prolapse of the left coronary cusp (Fig. **1a**), a 3-mm vegetation-like lesion on the left coronary cusp (Fig. **1b**), and severe eccentric AR with a vena contracta width of 7 mm, as demonstrated by color Doppler (Figs. **1c**-**e**). The left ventricular end-diastolic dimension (LVEDD) was 50 mm, and the left ventricular ejection fraction (LVEF) was 65%. The aortic root and ascending aorta diameters were within normal limits (sinus of Valsalva 35 mm, ascending aorta 32 mm).

### 2.1 Clinical Course

#### 2.1.1 Phase 1 (Acute Management)

Laparoscopic cholecystectomy was successfully performed with perioperative broad-spectrum antibiotic prophylaxis. The postoperative course was uneventful. Following supportive management with cardiotonic agents, diuretics, and potassium supplementation, a repeat TTE revealed a LVEDD of 46 mm and an LVEF of 63%. Laboratory studies showed pro-BNP, CRP, and erythrocyte sedimentation rate (ESR) levels of 2413 pg/mL, 4.8 mg/L, and 2.0 mm/h, respectively. The patient was subsequently discharged for outpatient follow-up.

#### 2.1.2 Phase 2 (Cardiac Surgical Intervention)

 Two months after the cholecystectomy, the patient reported recurrence of chest oppression and paroxysmal nocturnal dyspnea over the past 10 days, which had worsened in the last 4 days (NYHA class III). Follow-up TTE showed stable severe AR, but a further increase in the LVEDD to 56 mm, along with elevated pulmonary artery pressure (PASP 52 mmHg). Based on these findings, mechanical aortic valve replacement was indicated. Intraoperative examination identified prolapse of the left coronary cusp and vegetation on the left coronary leaflet. Histopathological examination demonstrated nonspecific inflammatory infiltration with negative microbial cultures. Early postoperative TTE confirmed normal prosthetic valve function without paravalvular leakage. The LVEDD subsequently decreased to within the normal range (46 mm) over two weeks. However, CRP levels remained persistently elevated at >50 mg/L for two months.

#### 2.1.3 Phase 3 (Complications)

At three-month follow-up, echocardiography revealed a trace paravalvular leak (regurgitant jet width: 4 mm), accompanied by a LVEDD of 49 mm and a LVEF of 62%. Four months postoperatively, multiple oral and scrotal ulcers prompted rheumatology consultation, leading to a definitive BD diagnosis. Immunosuppressive therapy with thalidomide and colchicine was initiated.

#### 2.1.4 Phase 4 (Terminal Event)

 Six months postoperatively, the patient developed acute dyspnea (NYHA class IV). TTE revealed a 6-mm paravalvular leak with concomitant left ventricular enlargement (LVEDD 54 mm). Ten days later, acute worsening of chest tightness and dyspnea necessitated emergency echocardiography, which showed partial dehiscence of the mechanical aortic valve prosthesis exhibiting flailing motion (Figs. **2a**-**d**) and paravalvular leakage (Figs. **2e**, **f**). Emergency reoperation confirmed valve dehiscence with paravalvular leaks at the left and right coronary sinuses. The surgical intervention comprised excision of residual annular tissue and the Bentall procedure. The patient required venoarterial extracorporeal membrane oxygenation (VA-ECMO) after failure to wean from cardio-pulmonary bypass, and ultimately succumbed to refractory cardiogenic shock and multiorgan failure. Pathological examination of the excised annular tissue revealed focal degeneration and necrosis with scant lymphocytic and neutrophilic infiltration (Figs. **3a**, **b**).

## 3 DISCUSSION

According to the 2014 International Criteria for Behçet's Disease (ICBD), the diagnosis of BD requires a score ≥4 based on ocular lesions (2 points), genital ulcers (2 points), oral ulcers (2 points), skin lesions (1 point), neurological manifestations (1 point), vascular manifestations (1 point), or a positive pathergy test (1 point) [3]. This patient's initial presentation, isolated AR without mucocutaneous lesions, highlighted a critical diagnostic gap. Notably, AR correlates with adverse cardiovascular outcomes, including increased mortality among BD patients [4]. BD patients undergoing valve surgery face substantially increased risks of paravalvular leakage (PVL) [5]. Therefore, early diagnosis and comprehensive evaluation of valvular abnormalities are essential for improving prognosis in BD.

Although the precise pathogenesis of BD-related AR remains incompletely characterized, genetic susceptibility, environmental triggers, and immune dysregulation are implicated. Pathologically, distinctive aortitis drives annular dilatation, sinus of Valsalva pseudoaneurysms, and direct leaflet inflammation [6]. Echocardiography is indispensable for detecting valvular abnormalities. An early study by Jae-Kwan Song *et al*. [7] documented aortic valve prolapse in BD patients with AR, often accompanied by perivalvular echo-free spaces or mass-like lesions. A recent study has further confirmed this observation, reporting that in BD patients with severe AR, 50% exhibit aortic valve prolapse, 20% show vegetation-like lesions, and a minority manifest aortic leaflet perforation or aneurysmal changes [8]. The echocardiographic findings in the present case were consistent with this. However, these manifestations are not specific to BD and can also occur in valvulopathies caused by other etiologies, such as infective endocarditis (IE). Indeed, the attending physician in this case initially considered acute AR secondary to IE, likely based on the echocardiographic findings, leukocytosis, and elevated serum CRP levels. However, subsequent blood cultures failed to identify any pathogens, and pathological examination of the specimen revealed no evidence of active infective endocarditis. Furthermore, studies have found that over half of BD patients with AR also have aortic root dilatation [8]. Although absent in this case, its presence strongly supports the BD etiology. This feature can aid in differentiating BD from IE, as their treatment strategies are fundamentally different. Unlike IE, BD-related valvulitis is inherently a sterile inflammation, rendering antibiotic therapy ineffective and underscoring the necessity of immunosuppressive treatment. Therefore, recognizing the characteristic echocardiographic features of BD-induced acute AR holds significant value in the differential diagnosis of unexplained acute AR.

Echocardiography plays a crucial role in prognostic assessment and monitoring of postoperative complications. Compared to AR secondary to degenerative or rheumatic etiologies, BD-associated valvulopathy confers significantly elevated risks of PVL, prosthetic valve dehiscence, recurrent regurgitation, and reoperation [9]. Preoperative characteristics, including redundant aortic valve tissue, ascending aortic dilation, or pseudoaneurysm, correlate with higher rates of recurrent AR following initial surgery [10]. To address these challenges, modified surgical techniques (*e.g*., comprehensive aortic root reinforcement or radical root replacement) may mitigate these risks by addressing the inherent fragility of inflamed tissues [11]. Perioperative immunosuppressive therapy is equally vital, as uncontrolled systemic inflammation may precipitate early or late prosthetic valve failure. Studies demonstrate that short-term preoperative immunosuppression partially controls acute inflammation, while long-term maintenance therapy is essential for reducing mortality and preventing postoperative complications [12]. Early diagnosis based on characteristic echocardiographic features, combined with aggressive surgical debridement of the aortic root and postoperative immunosuppression, is critical to improve clinical outcomes.

This case has highlighted the dilemma between standard surgical indications and the risks of operating on a patient with active, undiagnosed vasculitis. Surgery was indicated due to acute symptom exacerbation and progressive left ventricular enlargement [13]. However, it is noteworthy that the persistently elevated CRP level following aortic valve replacement, despite the absence of infectious symptoms, has provided a critical insight that was not sufficiently appreciated in the initial management. The sustained elevation of CRP should serve as a strong indicator, suggesting the possibility of an active non-infectious inflammatory process [14]. In this case, the elevated CRP level represented a serological manifestation of ongoing active vasculitis, reflecting the systemic inflammatory burden of BD. Although non-specific, this biochemical finding should have prompted a more thorough investigation into autoimmune etiologies prior to cardiac surgery. Recognizing the significance of unexplained inflammation might have guided earlier immunosuppressive therapy, which could potentially have improved the patient’s outcome.

Diagnosing BD remains challenging due to the absence of disease-specific biomarkers. Diagnosis heavily relies on clinical vigilance, particularly the recognition of characteristic mucocutaneous and systemic manifestations. These manifestations can be subtle, delayed, or absent in atypical cases. This case has exemplified a dilemma: isolated AR preceded mucocutaneous manifestations by months, causing critical delays. This has highlighted a key clinical principle as follows: unexplained AR with elevated inflammatory markers or valvulitis features (*e.g*., non-infectious vegetations) warrants rigorous occult BD screening, even without classic symptoms. Early rheumatology consultation and echocardiographer awareness of BD-specific patterns can enable timely immunosuppression. Multidisciplinary management thus becomes essential to avert catastrophic outcomes.

A high index of suspicion for BD is essential when evaluating acute aortic regurgitation accompanied by valve prolapse or non-infectious vegetations, especially in the presence of aortic root involvement and negative blood cultures, since cardiac involvement may be the initial and dominant manifestation of BD. In cases of unexplained valvulitis with persistent inflammation, cardiologists and cardiac surgeons should actively exclude occult BD through multidisciplinary collaboration with rheumatology. If surgery is unavoidable, a radical procedure, such as the Bentall operation, is preferred over simple valve replacement to minimize the risk of paravalvular leak resulting from tissue fragility. Whenever possible, surgery should be delayed until immunosuppressive therapy has controlled active inflammation. For rheumatologists, recognition of characteristic echocardiographic features is crucial to facilitate early diagnosis and guide medical therapy, which could play a critical role in improving surgical outcomes and long-term prognosis.

## CONCLUSION

The diagnosis of BD remains challenging owing to the absence of specific biomarkers and the occurrence of atypical presentations, including isolated AR presenting prior to mucocutaneous lesions. Recognition of characteristic echocardiographic features, namely aortic valve prolapse, non-infectious vegetation-like lesions, and aortic root dilatation, is critical for distinguishing sterile BD-related valvulitis from conditions, such as infective endocarditis, given the fundamental therapeutic divergence between immuno-suppression and antibiotics. BD-associated valvulopathy confers significantly elevated risks of postoperative complications, specifically paravalvular leak and prosthetic dehiscence. Therefore, early diagnosis guided by echocardiographic patterns is essential, necessitating modified surgical techniques that address tissue fragility and aggressive perioperative immunosuppressive therapy. Multidisciplinary management and rigorous screening for occult BD in cases of unexplained AR associated with inflammation are critical for improving the outcomes.

## Figures and Tables

**Fig. (1):**
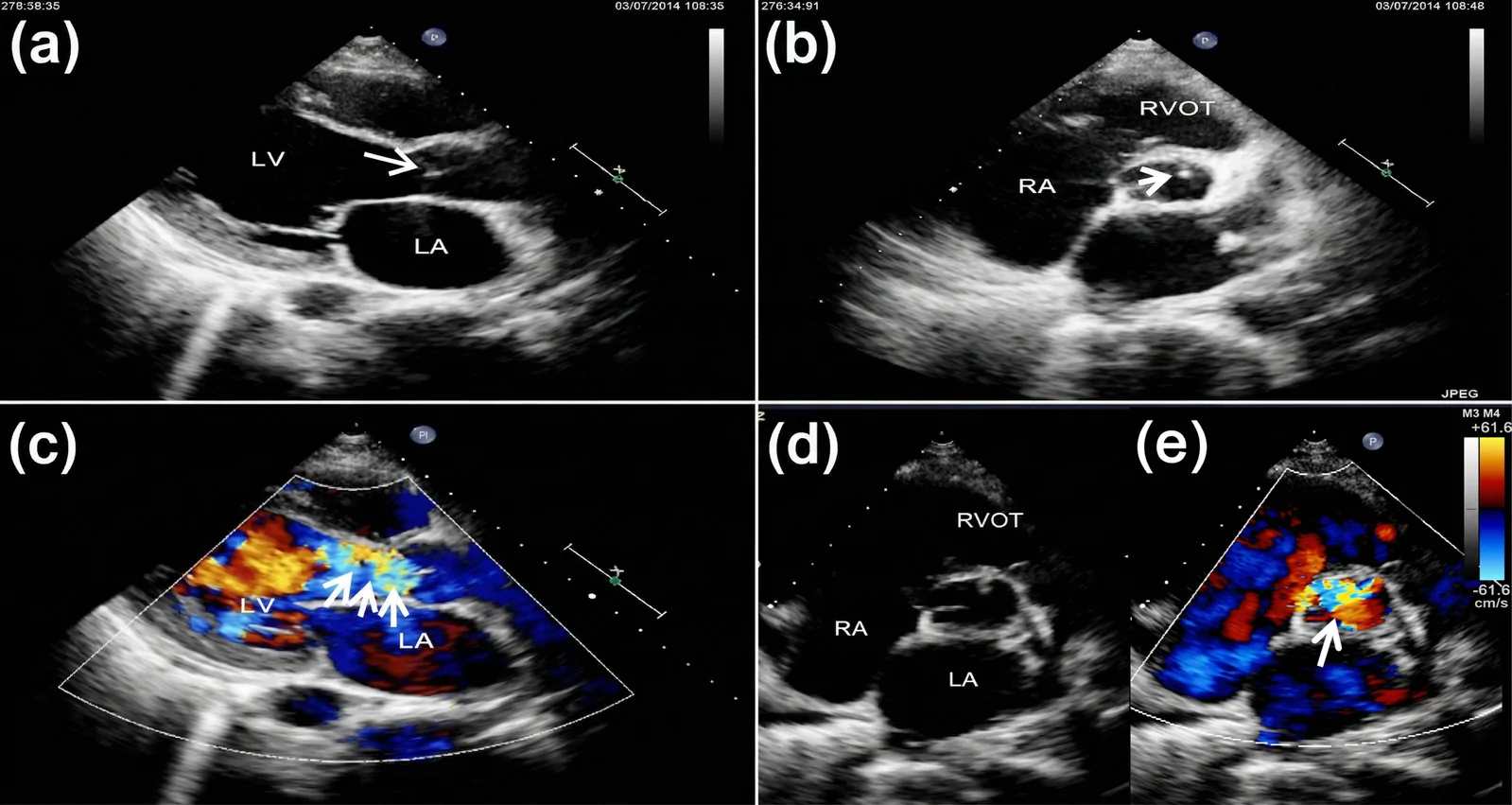
(**a**) Parasternal view TTE demonstrates malcoaptation of the aortic left and right coronary cusps, with mild prolapse of the left coronary cusp toward the left ventricular outflow tract (white arrow). (**b**) TTE reveals a 3-mm hyperechoic vegetation attached to the tip of the left coronary cusp (white arrow). (**c**-**e**) TTE shows severe eccentric regurgitation below the aortic valve during the diastolic phase (white arrows).

**Fig. (2):**
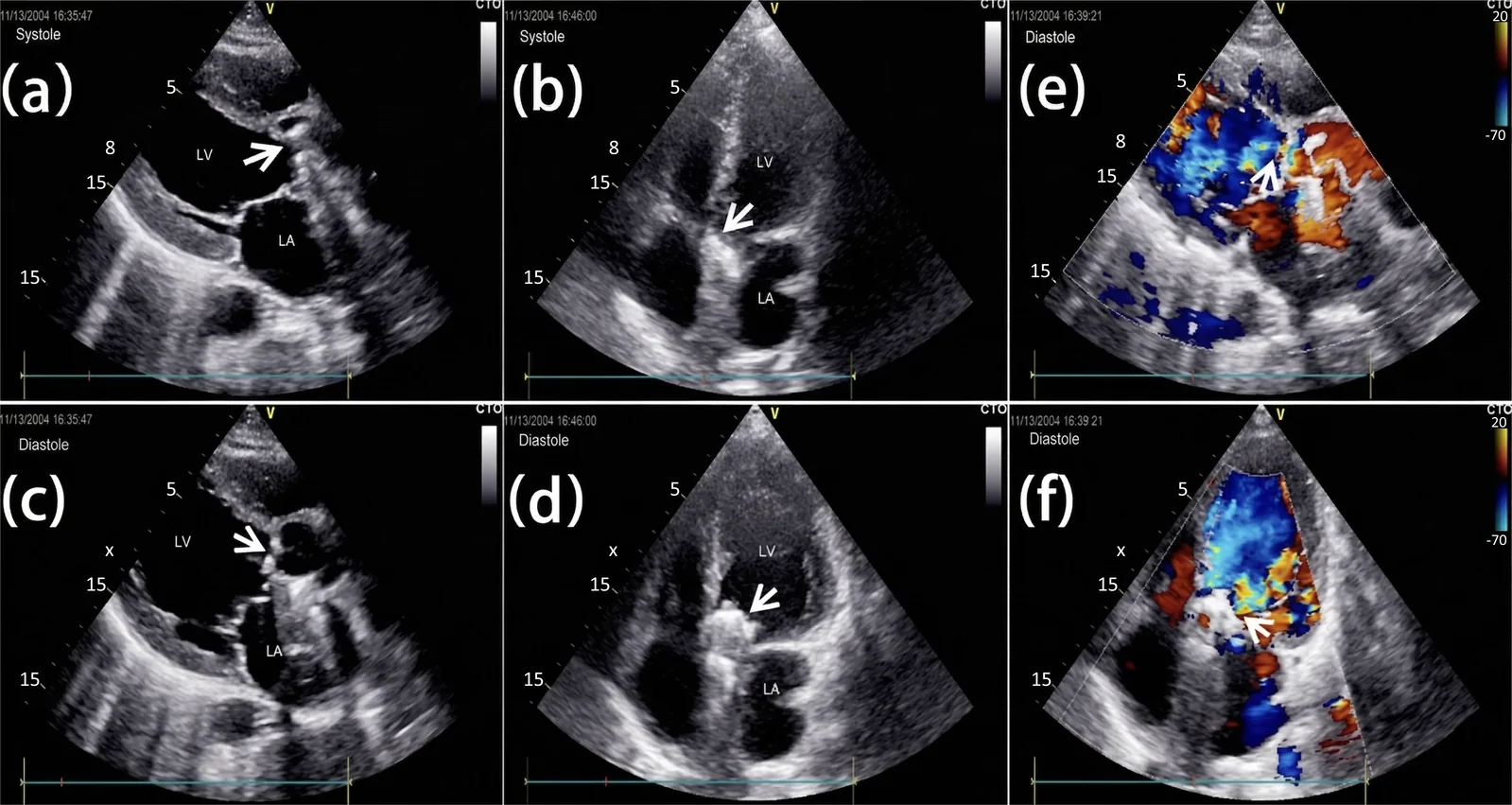
(**a**-**d**) Parasternal left ventricular long-axis and apical views on TTE demonstrate dislocation of the prosthetic mechanical aortic valve, with flailing motion throughout the cardiac cycle (white arrows). (**e** and **f**) Parasternal left ventricular long-axis and apical views on TTE reveal significant paravalvular regurgitation around the prosthetic mechanical aortic valve (white arrows).

**Fig. (3):**
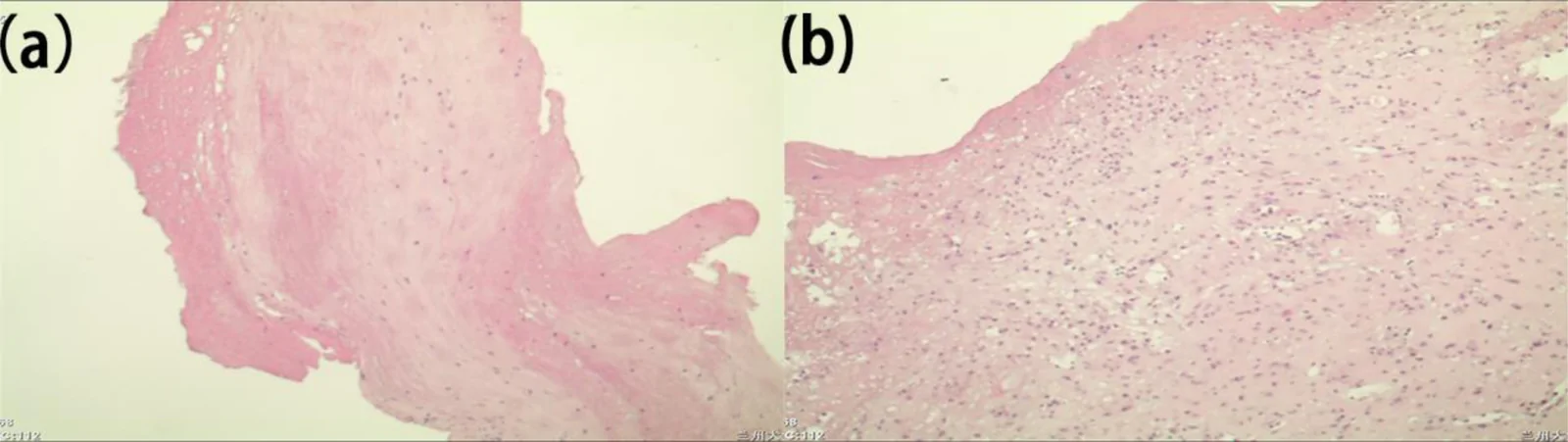
(**a** and **b**) The annular tissue exhibits focal areas of degeneration and necrosis, with scattered infiltration by lymphocytes and neutrophils.

## Data Availability

The data and supportive information are available within the article.

## LIST OF ABBREVIATIONS

AR: Aortic regurgitation
BD: Behçet's disease
CT: Computed tomography
ESR: Erythrocyte sedimentation rate
ICBD: International Criteria for Behçet's Disease
IE: Infective endocarditis
LVEDD: Left ventricular end-diastolic dimension
LVEF: Left ventricular ejection fraction
NYHA: New York Heart Association
PVL: Paravalvular leakage
TTE: Transthoracic echocardiography
VA-ECMO: Venoarterial extracorporeal membrane oxygenation

## STANDARDS OF REPORTING

CARE guidelines were followed.
